# c-MAF, a Swiss Army Knife for Tolerance in Lymphocytes

**DOI:** 10.3389/fimmu.2020.00206

**Published:** 2020-02-14

**Authors:** Claire Imbratta, Hind Hussein, Fabienne Andris, Grégory Verdeil

**Affiliations:** ^1^Department of Oncology, University of Lausanne, Lausanne, Switzerland; ^2^Laboratoire d'Immunobiologie, Université Libre de Bruxelles, Brussels, Belgium

**Keywords:** T cells, tolerance, c-Maf, interleukin-10, gut

## Abstract

Beyond its well-admitted role in development and organogenesis, it is now clear that the transcription factor c-Maf has owned its place in the realm of immune-related transcription factors. Formerly introduced solely as a Th2 transcription factor, the role attributed to c-Maf has gradually broadened over the years and has extended to most, if not all, known immune cell types. The influence of c-Maf is particularly prominent among T cell subsets, where c-Maf regulates the differentiation as well as the function of multiple subsets of CD4 and CD8 T cells, lending it a crucial position in adaptive immunity and anti-tumoral responsiveness. Recent research has also revealed the role of c-Maf in controlling Th17 responses in the intestine, positioning it as an essential factor in intestinal homeostasis. This review aims to present and discuss the recent advances highlighting the particular role played by c-Maf in T lymphocyte differentiation, function, and homeostasis.

## Introduction

The *Maf* (musculoaponeurotic fibrosarcoma) gene encodes the transcription factor c-Maf or MAF. Originally identified in natural musculo-aponeurotic fibrosarcoma of chickens infected with the replication-defective retrovirus AS42, the founding member of the Maf family, named v-Maf, was described as an oncogene (1–3). Using a probe containing the v-Maf sequence, its cellular counterpart, identified as c-Maf, was thereafter cloned from a number of vertebrate genomes (4). In addition to its function as an oncogene, c-Maf was soon found to regulate various cellular differentiation and developmental processes within tissues. In particular, c-Maf expression controls lens fiber cell differentiation, crystalline gene expression, as well as lens development (5–7). In neural tissue, c-Maf controls the expression of mechanoreceptors involved in touch sensation (8, 9). It also regulates the embryonic development of tubular renal cells (10) and the differentiation of chondrocytes during endochondral bone development (11–13). c-Maf plays a predominant role for the erythropoiesis that accompanies erythroblastic islands formation in fetal liver (14). In porcine and human pancreatic islets (15), c-Maf also regulates glucagon hormone production, thereby establishing pancreatic endocrine function (16). In line with the major contributions of c-Maf in developmental and physiological processes, mice lacking c-Maf are embryonically (14) or perinatally (5, 7) lethal depending on the type of C57BL/6 background. Some mice on the BALB/c background live to adulthood (10, 13).

In parallel to the discovery of the many roles of c-Maf within tissue development, c-Maf soon emerged as an immune regulator and was initially identified as a Th2 transcription factor. Similar to its function in tissue development, the role attributed to c-Maf within immune regulation broadened over the years and has extended to most, if not all, known immune cell types. While the role of c-Maf has also been studied within innate immune cell types (17–19) and B lymphocytes (20), we focus on c-Maf within T cell subsets, where c-Maf regulates the differentiation as well as the function of multiple subsets of CD4 T cells, lending it a crucial position in T cell immunity. Recent research has revealed the role of c-Maf in the control of intestinal Th17 responses by regulatory T cells, positioning it as an essential factor in regulatory T cell specification and, more broadly, the maintenance of intestinal homeostasis. This review aims to present and discuss the recent advances highlighting the particular role played by c-Maf in T lymphocyte differentiation, function, and homeostasis.

## The c-Maf Transcription Factor

**Figure 1:**
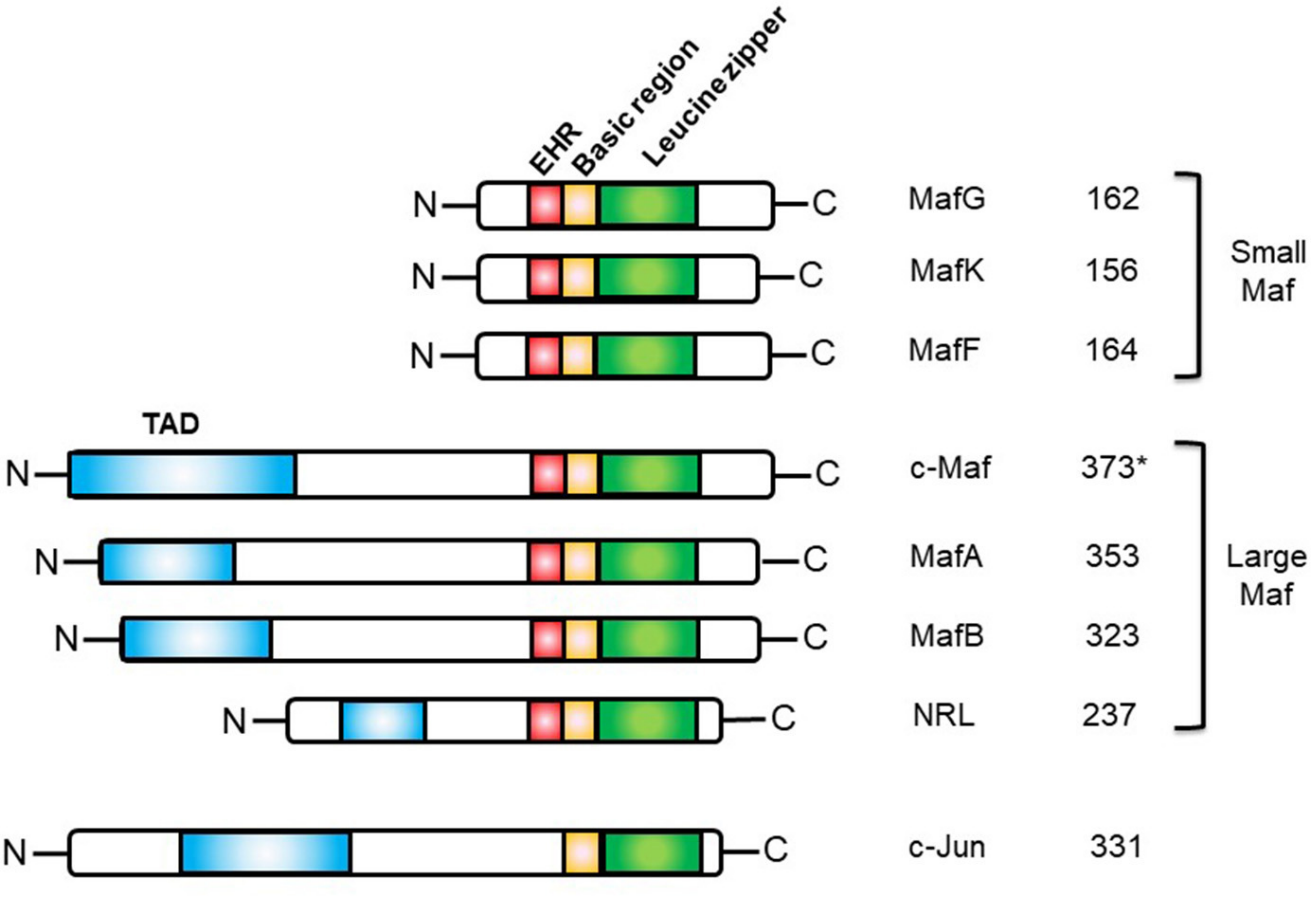
Structures of the human Maf family proteins and the c-Jun bZIP transcription factor. EHR, extended homology region; TAD, transactivation domain. *The short isoform (isoform 1) of human c-Maf is represented.

This basic leucine zipper (bZIP) transcription factor belongs to the AP-1 superfamily, which includes Fos, Jun, ATF, and CREB. The Maf transcription factor family is composed of 7 members divided into two subclasses: the large Maf proteins composed of MAFA/L-MAF, MAFB, MAF/c-Maf, and NLR (neural retina leucine zipper), and the small Maf proteins, MAFK, MAFG, and MAFF, which lack the amino-terminal transactivation domain. The Maf family of transcription factors harbors a unique and highly conserved basic region-leucine zipper (bZIP) structure (21). The basic regions of dimeric Maf factors allows them to recognize a palindromic sequence referred to as the Maf Recognition Element (MARE). This sequence is composed of a 7-bp TPA-Responsive Element (TRE) or a 8-bp cyclic AMP-Responsive Element (CRE) core region and a TGC flanking sequence bound by the Extended Homology Region (EHR), exclusively found in Maf proteins (22) (Figure 1). This long recognition sequence thus distinguishes the Maf protein family from other AP-1 family members and contributes to the important functions of the Maf proteins (23).

Thanks to their leucine zipper domain, Maf proteins can form homo- and heterodimers with other compatible bZIP proteins, such as Jun and Fos (24, 25). Maf proteins can also interact with other non-bZIP proteins including specific transcription factors, such as Sox family members (11).

Three isoforms exist for human c-Maf: a short form (373 amino acids), a medium form (383 amino acids), and a long form (30 amino acids more than the short form) of 38.5, 39.6, and 42 kDa respectively. In mice, only two isoforms have been reported: the long form (380 amino acids), called MAF-201, and the short form (370 amino acids). So far, distinct c-Maf products have not shown functional differences, but a potential functional specification cannot be excluded.

c-Maf is located on the chromosome 16q23.2 in humans and on chromosome 8 in mice (26, 27). c-Maf is translocated in 5–10% and/or overexpressed in 50% of multiple myelomas (MM) (28, 29). c-Maf overexpression in MM drives cyclin D2, integrin β7 and ARK5 expression and leads to proliferation, adhesion to bone marrow stroma cells, invasion, and migration of plasma cells (30). c-Maf is also highly expressed in over half of the angioimmunoblastic T-cell lymphomas (AITL) (30, 31). Transgenic overexpression of c-Maf in T cells regulates the same gene expression set as in plasma cells and induces T-cell lymphoma development in mice (30), therefore indicating that c-Maf is a bona fide oncogene contributing to the progression of hematological malignancies. c-Maf is also expressed by other cancers, such as renal or head and neck cancer, yet its expression is not systematically correlated with a bad prognosis (32).

## Induction of c-Maf in T Cells

c-Maf expression and activity are regulated at transcriptional, post-transcriptional, as well as post-translational levels. Transcription factors and RNA-mediated silencing control the amount of Maf transcripts, while phosphorylation and SUMOylation modify the activity, sub-cellular localization, and half-life of the protein.

In T cells, antigenic stimuli that modulate the stability of the *Maf* encoding mRNAs and/or the c-Maf protein may also induce the expression of transcriptional activators, or may cooperate with independent transcriptional stimuli, such as cytokine-driven STAT factors to induce c-Maf transcription. The selective use of those pathways by different stimuli and in distinct cell populations provides the potential for tailoring c-Maf expression to different circumstances.

### Transcriptional Regulation

**Figure 2:**
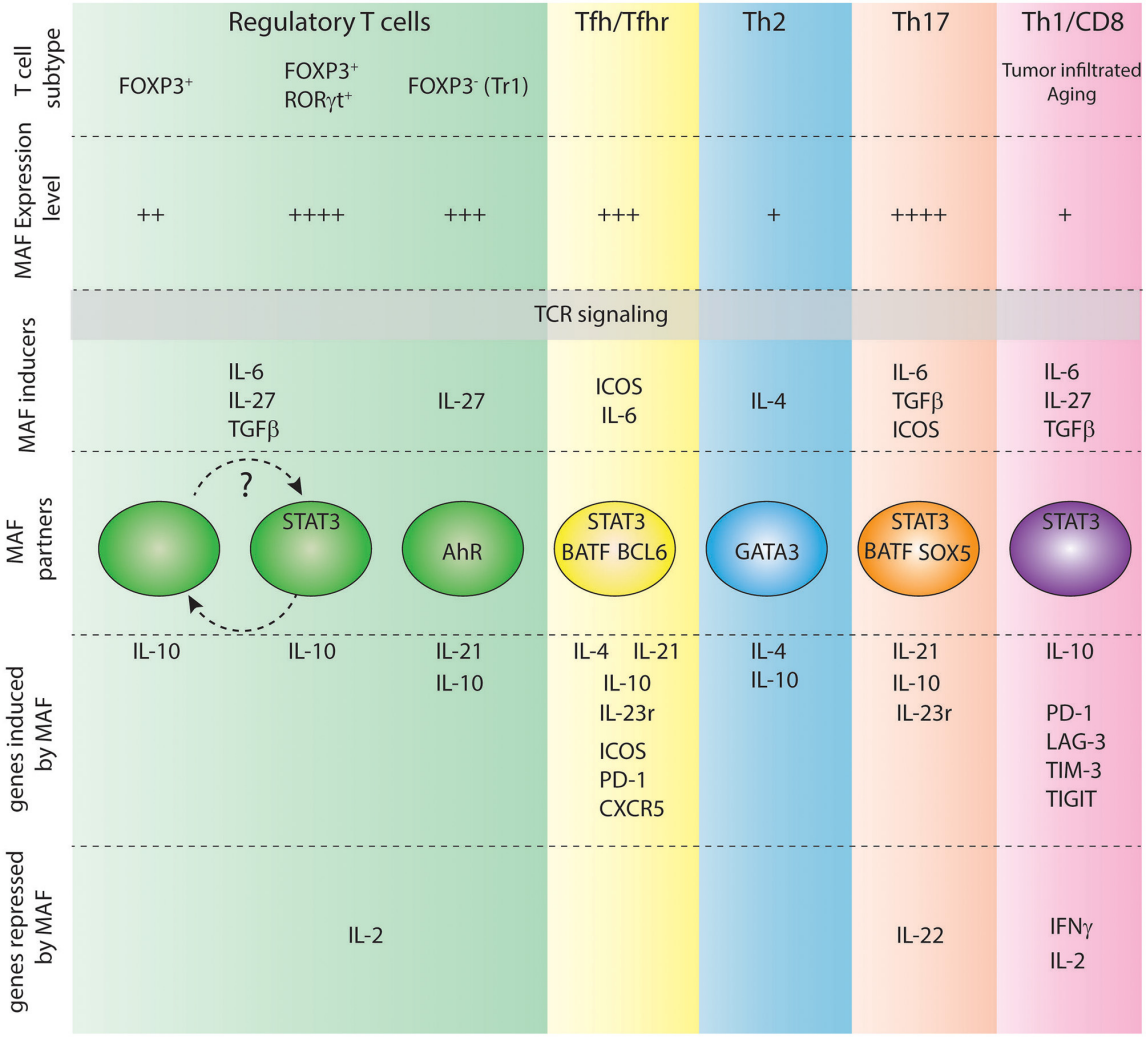
Role of c-Maf in various T cell subtypes. Level of expression of c-Maf, signals regulating its expression, partners of c-Maf and c-Maf target genes in each indicated T cell subtype are shown.

In T cells, TCR stimulation induces the transcription of the *Maf* gene. However, distinct additional stimuli are required to sustain the expression of *Maf*, such as co-stimulatory signals (33) or the presence of cytokines, including IL-4 (34), IL-6 (35), TGF-β (36), and IL-27 (37) (Figure 2).

The highest levels of *Maf* transcripts can be detected in Th17 and Tfh cells. During Th17 cell polarization, both TGF-β and IL-6 are required for maximum induction of c-Maf, which in turn depends on STAT3 phosphorylation, but not STAT1 or STAT6 activation (35, 36, 38, 39). Moreover, after IL-6 stimulation, STAT3 binds to the promoter region of *Maf* in CD4 T cells and transactivates *Maf* in a luciferase reporter gene assay (35), thus positioning STAT3 as an important STAT transcription factor for c-Maf expression in T cells.

c-Maf expression was initially thought to rely on the IL-4/STAT6 signaling pathway during Th2 cell differentiation, as ectopic expression of activated STAT6 in Th1 cells promoted c-Maf expression, along with Th2-specific cytokines and GATA3 expression (34). However, introducing GATA3 into STAT6-deficient T cells restored c-Maf expression, therefore suggesting an indirect role of STAT6 in c-Maf induction during Th2 differentiation (40). Of note, the IL-6/STAT3 signaling pathway is central to c-Maf expression during Th2 cell development (35, 41). In particular, Th2 cells express activated forms of STAT3 downstream of a STAT6-signaling pathway. Ablation of STAT3 in developing Th2 cells does not preclude GATA3 and IL-4 expression but selectively impairs c-Maf expression (41). This STAT6-to-STAT3 signaling pathway thus reconciles previous contradictory results concerning the role of STAT6 in c-Maf induction and further supports an indirect role of STAT6 in c-Maf expression.

The inducible co-stimulator (ICOS), expressed by activated T cells, promotes expression of c-Maf in murine Th2 cells and in both mouse and human Th17 cells, although the molecular mechanisms beyond this induction are still ill-defined (33, 42–44).

IL-27, a member of the IL-12/IL-23 heterodimeric family of cytokines produced by APCs, is also a potent inducer of c-Maf during Tr1 cell differentiation (45). Interestingly, IL-27 signals through STAT1/STAT3 has been shown to up-regulate ICOS expression, thus activating two independent pathways that might up-regulate c-Maf.

Prostaglandin E2 (PGE2), a pro-inflammatory lipid mediator abundant at inflammatory sites, has recently been shown to inhibit c-Maf expression in developing Tr1 cells (46). PGE2 did not affect STAT1/3 activation and its inhibitory effect was mediated through the EP4 prostaglandin receptor and cAMP signaling (46).

The expression of a c-Maf specific intergenic long non-coding RNA, called linc-MAF-4, in Th1 cells inhibits *Maf* transcription through the recruitment and activation of chromatin-modifying complexes, including the PCR2-associated histone methyltransferase, enhancer of zeste homolog 2 (EZH2), and the lysine-specific histone demethylase 1A (LSD1) (47). Up-regulation of linc-MAF-4 in human CD4 T cells is directly involved in the down-regulation of *Maf* expression and correlates with encephalitogenic Th cell differentiation and annual relapse rate in patients with multiple sclerosis (48).

### Post-transcriptional Regulation

c-Maf expression is tightly regulated by small non-coding microRNAs (miRNAs). In particular, *Maf* contains phylogenetically conserved miR-155 seed matches in its 3′-UTR. Luciferase reporter experiments confirmed that the c-Maf 3′-UTR is a direct target of miR-155 (49). MiR-155 is strongly expressed in activated T cells and genetic invalidation of miR-155 led to increased levels of c-Maf in T cell lines, thus positioning miR-155 as a major regulator of c-Maf expression *in vivo* (49). The role of miR-155 in suppressing c-Maf expression has been further extended to microglia cells during the response to CNS ischemia (50). c-Maf is also targeted by miR-143 and miR-365 in macrophages (51), and by miR-1290 in laryngeal carcinomas (52). However, expression of those miRNAs has not yet been reported in T lymphocytes.

### Post-translational Control of the Biological Activity of c-Maf

Upon TCR activation, the CARMA1-dependent activation of the IKK complex results in the phosphorylation of the N-terminus part of c-Maf, which is required for nuclear translocation and binding to the promoter of target genes (53). In particular, the T cell-specific deficiency of either CARMA1 or its substrate IKKβ strongly reduced the DNA binding activity of c-Maf without affecting c-Maf abundance. This IKK-mediated activation of c-Maf is independent of NF-kB activation (53). Loss of CARMA1/IKK signaling resulted only in a partial decrease of c-Maf phosphorylation, suggesting that c-Maf might be phosphorylated by multiple kinases. c-Maf is phosphorylated by the Ser/Thr glycogen synthase kinase 3β (GSK3) in human multiple myeloma cell lines and in the lens, leading to protein stabilization (54, 55). However, whether GSK3 exerts a similar role in T cells is difficult to evaluate as GSK3 inhibition increases expression of c-Maf in this context (56).

Tyrosine phosphorylation of c-Maf is also critical for its recruitment to the IL-4 and IL-21 promoters and for optimal cytokine production. Phosphorylation of c-Maf on tyrosine residues has been shown to be positively and negatively regulated by the TEC tyrosine kinase and the PTPN22 tyrosine phosphatase, respectively (57).

SUMOylation of c-Maf at the lysine 33 residue reduces its ability to bind the *Il4* promoter and decreases the transactivating activity of c-Maf in a luciferase reporter assay (58, 59). In addition, a recent report indicated that c-Maf SUMOylation is negatively correlated with *Il21* expression in CD4 T cells from diabetogenic NOD mice (60). Furthermore, transgenic expression of a SUMO-defective c-Maf selectively inhibited recruitment of Daxx/HDAC2 to the *Il21* promoter and enhanced histone acetylation mediated by CREB-binding protein (CBP) and p300. Thus, the SUMOylation status of c-Maf has a stronger regulatory effect on IL-21 than the level of c-Maf expression, through regulation of epigenetic mechanisms (60).

## Roles of c-Maf IN T Helper Cells

### Regulation of IL-10 Secretion in Multiple T Cell Subsets

Multiple roles have been attributed to c-Maf in distinct T cell subsets (Figure 2), revealing context-specific effects of this transcription factor. However, c-Maf positively regulates *Il10* expression in virtually all immune cells, including T cells, B cells, macrophages, and dendritic cells (36, 37, 61–64), suggesting a common regulatory function beyond distinct T cell subset-specific roles.

IL-10 is an essential anti-inflammatory cytokine that plays important roles as a negative regulator of immune responses to foreign or self-antigens and prevents excessive inflammation during the course of infection [reviewed in (65–67)].

Exploring the role of c-Maf in three different disease models, each characterized by the predominant activity of a different T helper cell subset [malaria—Th1 cells; allergy to house dust mite—Th2 cells; experimental autoimmune encephalitis (EAE)—Th17 cells], Gabryšová et al. recently reported that *Il10* expression was significantly lower in the absence of c-Maf, in T helper cells across all three diseases (67). The combined evidence of open chromatin (ATAC-seq analysis) coincident with binding of c-Maf to the *Il10* locus (ChIP-seq analysis) confirmed c-Maf as a direct positive regulator of *Il10 in vivo* in distinct Th cell subsets (67).

c-Maf binds to consensus MARE motifs in the *Il10* promoter (36, 37). Although c-Maf can transactivate *Il10* by itself to some extent, c-Maf alone is not sufficient to induce optimal *Il10* expression in T cells (36, 37). Robust IL-10 expression requires interaction with additional transcriptional regulators that vary among T cell subsets. c-Maf cooperates with the aryl hydrocarbon receptor (AhR) to induce IL-10 in regulatory type 1 (Tr1) cells (37). AhR expression is mainly driven by TGF-β (68) and is not expressed in Th1 cells, in which fine-tuning IL-10 expression mostly relies on the interaction of c-Maf with Blimp-1 (64). IL-10 expression in Th2 cells relies on transcription factors STAT6, GATA3, and IRF4 (69, 70) but whether these factors interact directly with c-Maf awaits further investigation.

Thus, the activity of c-Maf on the *Il10* enhancer might not only depend on the accessibility of its motif but also on the nature of the other transcription factors that co-bind to that enhancer. In other words, *Il10* expression in Th cells relies on several transcriptional programs that, together with c-Maf, are able to integrate various signals from the environment in order to fine-tune this critical immunosuppressive cytokine.

In addition to a direct positive transcriptional regulation of *Il10* expression, c-Maf also provides a common mechanism for a negative regulation of IL-2 signaling *in vivo* in models of Th1, Th2, and Th17 responses (67).

However, although c-Maf uses common mechanisms of gene regulation in distinct cell subsets, the net outcome of c-Maf-deficiency is different in each cell type, thus indicating that c-Maf has context-specific effects on the immune response, over and above its effects on IL-10 and IL-2 signaling.

### Context-Specific Effects of c-Maf on Th Cell Subset Function

#### Th1/Th2 Cells

The cross-regulation between Th1 and Th2 cells is mediated, in part, by the transcription factors that they express. c-Maf was first described as a Th2-specific gene that induces *Il4* gene transcription via direct binding to the *Il4* but not the *Il5* or *Il13* locus (71). Transcription factors GATA3, STAT6, and NFAT can synergize with c-Maf to regulate IL-4 expression in Th2 cells (72–74). Moreover, overexpression of c-Maf skews the immune response toward a Th2 response (75).

Although these pioneer studies concluded to a pro-Th2 role of c-Maf, normal levels of IL-13 and IgE were observed in c-Maf-deficient mice (72). Contrary to a pro-Th2 effect of c-Maf on the immune response, increased Th2 lung pathology, associated with higher numbers of eosinophils in bronchoalveolar lavage fluids, was observed in the HDM allergy model despite a decreased expression of *Il4* in T-cell specific c-Maf deficient mice (67). Of note, cells producing both IL-4 and IL-10, but not IL-4^+^ IL-10^−^ Th cells, were lost in this allergy model, in keeping with increased pathology. Thus, although c-Maf can activate the *Il4* promoter, its net effect over the Th2 inflammatory response is mainly inhibitory.

In contrast with data obtained in naïve Th cells, ectopic expression of c-Maf in mature Th1 cells did not grant them the ability to produce IL-4, but did decrease their production of IFN-γ (75). In a recent study, chronically activated Th1 cells that were cultured with IL-4-producing Th2 cells up-regulate *Maf* expression (76). These cells were shown to down-regulate *Ifng* expression and express a dampened Th1 encephalitogenic cell capacity *in vivo*, despite normal expression of T-bet. Blockade of IL-4R signaling inhibited c-Maf expression in Th1 cells, suggesting that c-Maf may act downstream of IL-4R signaling to inhibit IFN-γ production in chronically activated Th1 cells (76). Thus, IL-4-driven expression of c-Maf in Th1 cells contributes to a transcriptional regulation program dampening their pathogenic immune response through altered cytokine profile.

In the malaria model, c-Maf-depletion led to greater acute-phase pathology, associated with enhanced expression of *Tbx21* and production of IFN-γ (67). This suggests a wider role of c-Maf, i.e., dampening expression of the master transcription factor T-bet, in this experimental context. However, no direct binding of c-Maf to the *Tbx21* locus was observed in the malaria model, indicating that c-Maf could regulate the expression of *Tbx21* through indirect mechanisms. Gabryšová et al. showed that the chromatin landscape of Th1 cells is remodeled by c-Maf. They identified a strong enrichment of the Runx transcription factor-binding site in the remodeled loci and further observed increased Runx expression in c-Maf-deficient Th1 cells. Given the reported effects of Runx factors on IFN-γ production (77), it is tempting to postulate that c-Maf dampens Th1 cell differentiation at least partially via repression of Runx expression. Yet functional validation of this c-Maf/Runx control over Th1 pathology still awaits experimental testing.

Those c-Maf mediated changes in the chromatin landscape were not observed in the context of Th2 or Th17 cell pathologies, again showing that the role of c-Maf varies widely depending on T helper cell type.

#### Follicular Helper T Cells

Follicular helper T cells (Tfh) are key regulators of T cell-dependent long-term humoral immunity (78). Tfh cells express BCL6, a transcriptional repressor considered as the critical master regulator of Tfh cell development *in vivo* (79, 80), and also constitute the major source of IL-21, a cytokine necessary for IgG class-switch recombination and antibody affinity maturation (81).

Using retroviral ectopic expression of c-Maf or BCL6 in *in vitro*-derived human Tfh cells, Kroenke et al. first reported that c-Maf and BCL6 regulate distinct features of Tfh cell functions, with BCL6 required for Tfh cell development and c-Maf for promoting IL-21 secretion (82). However, recent data have shown that c-Maf is expressed early during Tfh cell differentiation and is critical for Tfh cell development *in vivo* (83). This is in agreement with the finding that Tfh cell differentiation strongly relies on ICOSL/ICOS and IL-6/STAT3 signaling, two pathways known to induce the expression of c-Maf (35, 84–86).

The relative roles of c-Maf, BCL6, and other transcription factors in initiating and maintaining Tfh cell are still poorly defined. As c-Maf-bound genes in Tfh cells hardly correlate with genes bound by BCL6 or Ascl2 (87, 88), it is tempting to speculate that cooperation between c-Maf and BCL6/Ascl2 is required to reach complete Tfh cell fate through orchestration of distinct sets of genes. Although c-Maf does not regulate *Bcl6* transcription, a defect in BCL6 expression in CD4 T cells was observed in the absence of c-Maf, thus suggesting that c-Maf could contribute to BCL6 expression in developing Tfh cells (83).

Beside its role in Tfh cell development, c-Maf is also required for adequate IL-4 and IL-21 production through transactivation of the *Il4* and *Il21* promoters (82, 89, 90). In particular, Sahoo et al. reported that c-Maf promoted IL-4 secretion in Tfh cells through both direct binding to the CNS2 region in the *Il4* locus and via induction of IRF4, thus revealing a distinct role of c-Maf in IL-4 secretion between Th2 and Tfh cell subsets (90).

#### Th17 Cells

**Figure 3:**
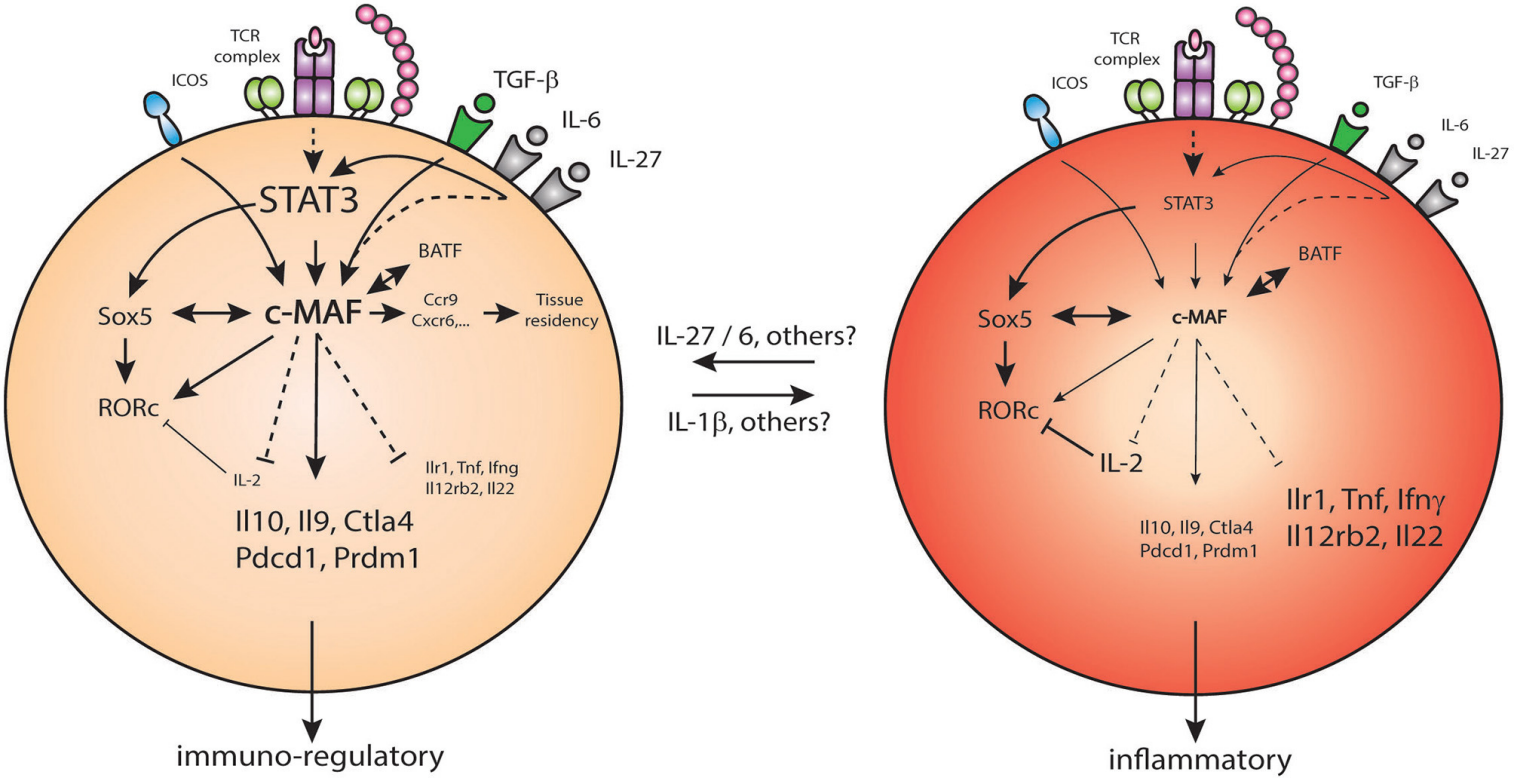
c-Maf regulates pro/anti-inflammatory properties of Th17 cells. Under the control of STAT3, c-Maf level in Th17 cells leads to differential expression of pro-inflammatory genes (*Ilr1, Tnfa, Ifng*,…) and immune-regulatory genes (*Il10, Ctla4, Pdcd1*,…) in Th17 cells.

c-Maf is highly expressed in Th17 cells and impacts several important aspects of their differentiation and function (33, 36, 39, 61, 64, 67, 91) (Figure 3). It physically associates with the transcription factor Sox5 and, together, they bind and activate the promoter of *Rorc* in conventional CD4 T cells. The Maf-Sox5 interaction thus controls Th17 development via the induction of RORγt as downstream targets of STAT3 (92). c-Maf also positively regulates certain loci in Th17 cells, including several genes known for controlling inflammation (e.g., *Il9, Lif*, *Il10*) (33, 37, 82, 89). c-Maf binds the *Il21* promoter, inducing the production of IL-21 which subsequently sustains Th17 expansion (83) and stabilization through IL-23R expression (33). Some evidence also suggests that c-Maf-induced IL-21 secretion could trigger a positive feedback loop by activating STAT3, thus further promoting c-Maf and leading to the development of memory Th17 cells (93). Sato et al. suggested that c-Maf can also directly transactivate the *Il23r* gene which contains a MARE-like sequence (93). However, the relation between IL-23R and c-Maf is still controversial as it was recently reported that c-Maf downregulates *Il23r* expression in a subset of memory Th17 cells (91). c-Maf has been shown to act as a repressor of *Il2* in CD4 T cells, which indirectly amounts to increased Th17 differentiation in EAE models. Indeed, T cell specific c-Maf deficiency led to an improvement of the disease through increased IL-2 production and decreased Th17 differentiation (67).

c-Maf also acts as a global negative regulator of genes associated with Th17 function. This was demonstrated by an exhaustive study of the regulatory network for Th17 cell specification, where c-Maf attenuates the expression of pro-inflammatory loci (e.g., *Rora, Runx1, Il1r1, Ccr6, Tnf*) and repressing genes belonging to pathways regulated by other core transcription factors. c-Maf represses IL-22, but not IL-17A production, in a TGF-β-dependent way. It binds to the *Il22* promoter and blocks the positive transcriptional effects of other Th17 transcription factors on the *Il22* locus, including RORγt and BATF (39).

Importantly, c-Maf maintains tolerance through the regulation of IL-10, as described above. In Th17 cells, c-Maf regulates the balance between the differentiation toward inflammatory or anti-inflammatory Th17 cells (91). c-Maf controls this balance by binding to enhancers or putative enhancers already available in the general landscape of Th17 cells but not through the direct binding to promoters (91). It promotes an immuno-regulatory program (*Il10, Ctla4*) while repressing pro-inflammatory associated genes (*Ifng, Il22, Il12rb2*) (91). Interestingly, c-Maf also promotes the expression of genes associated with memory Th17 cell tissue-residency, such as *Ccr9* and *Cxcr6* (91).

The environmental cues guiding the transition between inflammatory and anti-inflammatory Th7 cells are still not clearly established. The presence of inflammatory cytokines, such as IL-1β, favors an inflammatory state, as described in Aschenbrenner et al. (91), without directly affecting the level of c-Maf in the cell. The levels of STAT3 activating cytokines, such as IL-6, IL-27, or IL-10, may have the opposite effect and push Th17 cells toward a more anti-inflammatory phenotype through c-Maf induction (36, 91). Altogether, c-Maf plays a critical role in Th17 differentiation and functions by balancing the inflammatory properties of these cells, and by doing so, it plays a critical role in the regulation of tolerance/inflammation.

#### Regulatory T Cells (Treg) and Gut Tolerance

Regulatory type 1 (Tr1) cells have emerged as an important subset of T cells with strong immunosuppressive properties but which does not express master transcription factor Forkhead box 3 (Foxp3), contrary to regulatory T cells (Tregs). The protective role of Tr1 cells has been shown in numerous contexts, such as autoimmunity, colitis, graft-versus-host disease, and tissue inflammation (94). The anti-inflammatory effects of Tr1 cells mainly rely on their ability to produce high amounts of IL-10. The cytokine IL-27 is known to promote the expansion and the differentiation of Tr1 cells through the induction of transcription factor c-Maf and co-stimulatory receptor ICOS. In these cells, c-Maf physically interacts with AhR to transactivate the *Il10* and *Il21* promoters (37, 45, 95). Of note, the expression of IL-21 further sustains c-Maf expression in Tr1 cells, as a feed-forward loop, thus highlighting the major role of c-Maf, both in the induction and the stabilization of the Tr1 cell fate (37, 95).

In Foxp3^+^ Tregs, c-Maf is expressed at various levels. The fraction of c-Maf^+^ cells found in Tregs depends on the organ studied. In the thymus, spleen, mesenteric lymph nodes or lungs, 5–40% of Foxp3^+^ Tregs express c-Maf. Strikingly, a large proportion, ranging from 60 to 80%, of Foxp3^+^ Tregs express c-Maf in the gut (38, 96–99). In lymphoid organs, c-Maf expression is restricted to effector Tregs (eTreg), which express high levels of CD44 and low levels of CD62L (96, 98, 99). c-Maf expression is also induced when naïve Tregs are stimulated *in vitro* with anti-CD3 and anti-CD28 antibodies (96).

Distinct Treg populations adopt specialized phenotypes by co-expressing Foxp3 and lineage-defining transcription factors in response to tissue- or inflammatory-driven signals (100). In particular, RORγt^+^ Tregs represent a subset of Tregs that develops in the intestinal tissue of naïve mice in response to signals arising from a complex microbiota. This subset of peripherally induced Tregs has been shown to efficiently protect from intestinal immunopathology in different colitis models (101, 102) and to mediate immunological tolerance to the gut pathobiont *Helicobacter hepaticus* (38). RORγt^+^ Tregs express high levels of c-Maf and genetic ablation of c-Maf leads to a severe defect in the development of this subset (38, 96–99).

Numerous studies published in the last couple of years highlighted the critical role of c-Maf in the regulation of gut homeostasis. As mentioned above, c-Maf promotes IL-10 production in distinct T cell subsets. Using IL-10 reporter mice, Neumann et al. recently showed that IL-10 production by Tregs was strictly associated with c-Maf expressing Tregs (96). IL-10 is critically involved in regulating intestinal homeostasis as its total invalidation (103) or T cell specific inactivation (104–107) led to the development of a strong colitis in mice. IL-10 regulation therefore underlies a pivotal aspect of c-Maf control over gut homeostasis. Of interest, c-Maf regulates the development of RORγt^+^ Tregs, which are the major source of IL-10 in this organ (38, 97). Thus, c-Maf might control IL-10 production in the intestine both directly, through regulation of *Il10* gene transcription, and indirectly, via the differentiation of a high IL-10-producing Treg subset.

Genetic ablation of c-Maf also impairs the differentiation of follicular regulatory T cells (Tfr) in Peyer's patches (98). Tfr cells co-express both Tfh (CXCR5, PD-1, Bcl6) and Treg (Foxp3, CTLA4) markers and represent a regulatory T cell subset that controls the activity of Tfh cells. In the intestine, Tfr cells foster microbiota diversity via the regulation of symbiotic bacteria-specific IgA affinity (108). However, the functional consequences of Tfr cell deficiency in the gut homeostasis of mice harboring c-Maf-deficient Tregs requires further investigation.

Although the control exerted by c-Maf in gut homeostasis was clearly established by different groups, the extent of the c-Maf deficiency-driven inflammation varied drastically in distinct studies. Xu et al. reported that c-Maf inactivation in Tregs (Maf^ΔTreg^) induced a colitis with the apparition of a rectal prolapse in a third of the mice (38). This phenotype was observed after infection of the animals with the pathobiont *Helicobacter hepaticus*, but was much more reduced in the absence of infection. The same mouse model did not develop spontaneous colitis, and only showed mild signs of immune cell infiltration and colon tissue destruction in other studies that were not using the pathobiont (96, 97). Surprisingly, Maf^ΔTreg^ mice were even protected from acute DSS induced colitis by an increased production of IL-17 and IL-22 (96). Lastly, c-Maf inactivation in all T cell compartments (Maf^ΔT^) resulted in a spontaneous strong onset of colitis (97).

**Figure 4:**
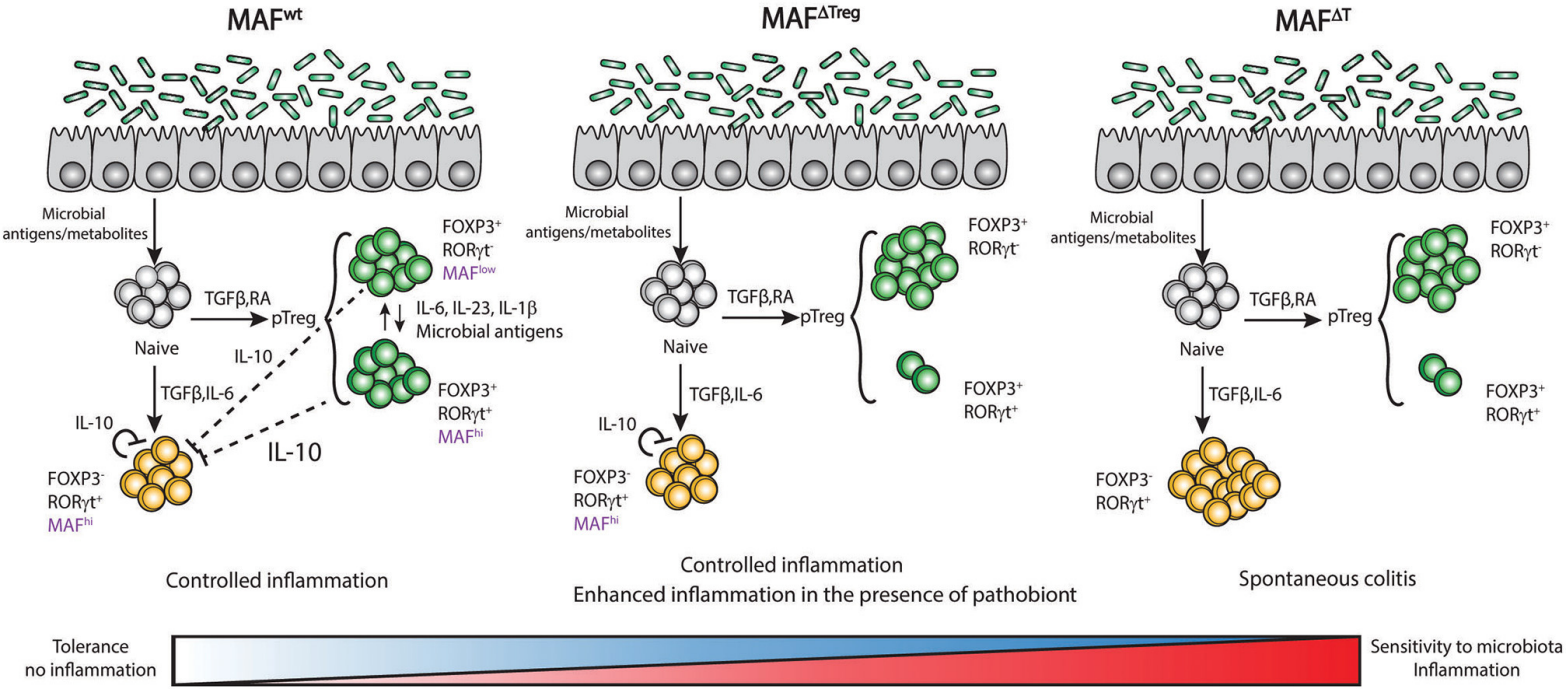
Expression of c-Maf in T cells regulates tolerance/sensitivity to microbiota in the gut. Expression of c-Maf in regulatory T cells (Foxp3^+^ RORγt^−^ or Foxp3^+^ RORγt^+^) as well as in Th17 cells (RORγt^+^ Foxp3^−^) regulates the expression level of IL-10 in the colon and is central in the regulation of tolerance to gut microbiota.

This gradation in colitis development might be related to subtle differences in the microbiota composition of mice bred in distinct animal facilities and to a gradient of inflammation induced by the presence or not of certain pathobionts. But it might also reflect the role of c-Maf in multiple T cell subtypes in the gut (Figure 4).

The absence of RORγt^+^ Tregs in the gut of Maf^ΔTreg^ mice strongly decreases the threshold for the development of colitis. However, unlike Maf^ΔTreg^ mice, RORγt^ΔTreg^ mice do not spontaneously develop colitis, suggesting that c-Maf has a more substantial role than RORγt in the function of intestinal Tregs. *Il10* transcripts are still detectable in the colon of Maf^ΔTreg^ mice (97) and Imbratta et al. recently reported that Th17 cells from the colon express high levels of c-Maf and can produce IL-10. In Maf^Δ*T*^ mice, this additional source of IL-10 is lost and the threshold for the development of colitis decreases further. This phenotype is lost when the animals are treated with antibiotics, further confirming that the microbiota plays a critical role in the development of colitis, probably through the induction of c-Maf and the development of RORγt^+^ Tregs in the colon (97). We can thus hypothesize that the presence of the microbiota induces a level of inflammation that triggers c-Maf expression in both intestinal Tregs and Th17 cells. c-Maf counterbalances this inflammation and promotes resistance to pathobionts through the production of IL-10 and/or the development of RORγt^+^ Tregs (Figure 4).

c-Maf inactivation strongly impaired IL-10 production in both Helios positive and negative Tregs in distinct organs, showing that c-Maf also regulates IL-10 production in Tregs of thymic and peripheral origin (96). However, ablation of c-Maf in the Treg or other T cell compartments did not result in systemic autoimmune disease, nor did it disturb conventional and regulatory T cell homeostasis in lymphoid organs, and c-Maf-deficient Tregs retained their *in vitro* suppressive capacity (38, 96, 98). Thus, although the precise role of c-Maf in the suppressive capacity of Tregs in distinct pathophysiological settings awaits further investigation, it seems that this transcription factor is a major player in colon homeostasis and that it drives immunological tolerance to gut pathobionts, thereby restraining inflammatory bowel disease.

## Role of c-Maf in Tumor Infiltrating CD8 T Cells

c-Maf is not expressed in CD8 T cells at steady state. The expression of c-Maf in CD8 T cells was first described in tumor infiltrating lymphocytes (TILs) obtained from a mouse melanoma model and from melanoma patient (109). Overexpression of c-Maf in CD8 T cells leads to a strong repression of IFN-γ and IL-2 production, and to an increased expression of genes associated with T cell exhaustion, i.e., a dysfunctional state of T cells observed during chronic infections or in TILs (110, 111). These genes include *Bcl6, Pdcd1* (PD-1), *Stat3*, and *Il10*, among other genes that are also described to be regulated by c-Maf in other T cell subtypes. When c-Maf was knocked-out in tumor specific CD8 T cells, those cells had a much higher capacity to restrain tumor growth through increased IFN-γ production and increased survival (109). Another study using B16 melanoma in mice confirmed that c-Maf was a major inducer of exhaustion associated genes, in cooperation with *Prdm1* (Blimp-1). These two transcription factors had a compensative effect for the regulation of the transcription of many inhibitory receptors, such as PD-1, TIGIT, TIM-3, or LAG-3 (112). The double knockouts for c-Maf and Blimp-1 led to a better control of B16 growth, compared to single knockouts or WT mice (112).

Tc17 cells are a rather rare subtype of CD8 T cells found in gastrointestinal cancers as well as other diseases ranging from fungal infection to bacterial colonization of the skin and response to influenza (113). A study on mouse and human Tc17 cells showed that c-Maf and RORγt were both essential for the development of these cells (113). Similar to what is observed in Th cells, c-Maf expression in CD8 T cells is associated with increased tolerogenic/non-inflammatory functions, a mechanism, which is hijacked by the tumor microenvironment to favor immune escape and tumor development.

## Role of c-Maf in Innate Lymphoid Cells and γδ T Cells

Innate lymphoid cells (ILCs) are tissue-resident cells which lack antigen specificity and are preprogrammed for effector function, poising them for rapid cytokine production and for tissular immune priming. Similar to T lymphocytes, ILCs can be classified into three specialized functional subclasses, characterized by the expression of lineage-defining transcription factors and effector cytokines (114). ILC1s are defined by T-bet (*Tbx21*) and IFN-γ production. ILC2s are characterized by their high GATA3 expression and IL-5 and IL-13 secretion. ILC3s are mainly located in the intestinal lamina propria and are defined by RORγt expression. They play a crucial role in maintaining epithelial barrier integrity by expressing large amounts of IL-22 and IL-17A (115). While c-Maf has been shown to be expressed early in ILC specification (116), it is preferentially expressed in intestinal ILC3s, particularly in CCR6^−^ NKp46^+^ cells (17, 117).

ILCs show functional and phenotypic plasticity in response to the environment (118). This is exemplified by the ability of ILC3s to upregulate T-bet, lose RORγt expression, and convert to IFN-γ-producing ILC1s, also called “ex-ILC3s,” which are involved in intestinal pathology (119–121). Recent analysis of ILC transcriptional regulatory networks showed that c-Maf regulates the ILC3/ILC1 balance in the intestine (18). c-Maf directly promotes ILC3 identity by upregulating canonical type 3 c-Maf signature genes, common to NKp46^+^ ILC3s, CCR6^+^ ILC3s, IL-17-producing γδ T cells, and RORγt^+^ Tregs (17). Moreover, c-Maf indirectly sustains ILC3 identity by inducing CD127 expression, which strengthens the IL-7-dependent promotion of RORγt expression in ILCs (17). In turn, c-Maf directly represses ILC1 conversion by binding to the *Tbx21* locus and inhibiting T-bet expression, and by restraining the type 1 chromatin accessibility landscape (17). Interestingly, this direct binding of c-Maf at the *Tbx21* locus was not observed in Th1 cells and seems to be ILC-specific (67).

IL-17-producing RORγt^+^ γδ T (Tγδ17) cells are innate-like γδ T cells, functionally preprogrammed to produce IL-17, positioning them as the main IL-17-producer in the steady state intestine and in various inflammatory diseases (122, 123). A recent study showed that the collaboration between c-Maf and RORγt is required for Tγδ17 induction and maintenance (19). In a similar dynamic to that observed in ILC3s, c-Maf sustains Tγδ17 differentiation both by supporting activating locus modifications and by antagonizing TCF1 accessibility, a negative regulator of Tγδ17 differentiation.

c-Maf therefore emerges as a global type 3 innate cell insulator, which sustains the expression type 3 genes, while constraining the acquisition of a type 1 phenotype by directly repressing type 1 genes, such as *Tbx21* and *Tcf7*, and by restraining chromatin accessibility. This feature distinguishes type 3 innate-like lymphoid cells from the adaptative Th17 cell subset, in which lineage-defining transcription factor expression and chromatin remodeling are regulated indirectly by c-Maf through negative regulation of the IL-2 signaling (67). c-Maf thus seems to occupy a higher position in the regulatory network controlling type 3 innate cells compared to that of Th17 cells.

## Perspectives: Manipulating c-Maf Expression to Treat Patients?

In all T cell subtypes, c-Maf plays an important role in regulating tolerance and homeostasis. Targeting c-Maf expression or function could be a good opportunity to enhance or repress a given immune response. As c-Maf is overexpressed in 50% of multiple myeloma, several laboratories developed strategies to find some potent inhibitors, which could be used to enhance the immune response, in an anti-cancer setting for instance. USP5 is important to regulate the degradation of c-Maf (124). Molecules targeting this pathway, such as Mebendazole, showed some potent anti-myeloma activity by inhibiting the USP5/c-Maf axis (125). Another possible way to inhibit c-Maf activity is by preventing its dimerization. This could be achieved by the design of specific peptide able to destabilize homodimers of c-Maf (126). An older study described that glucocorticoids are potential inhibitors of c-Maf in multiple myeloma (127). However, using these compounds to manipulate the immune system is rather inappropriate, as glucocorticoids strongly dampen it. Lowering c-Maf expression at the cellular level before T cell transfer is also one possibility, with the use of miRNA or similar technologies.

Increasing c-Maf expression in Tregs would be of crucial interest to treat autoimmune diseases, especially in the gut where c-Maf is important in several T cell subtypes. The c-Maf expressing RORγt^+^ Foxp3^+^ Treg population is also present in human colon but do not show any variation in proportion of total CD4 T cells when comparing colon from patients with Crohn's disease and healthy tissues (128). How to manipulate and increase c-Maf expression and RORγt^+^ Tregs remains a challenge that requires further studies. The microbiota appears to play a central role in the regulation of this regulatory population (97, 101, 128, 129) and manipulating c-Maf expression might have dramatic impact on microbiota equilibrium. Thus, a better understanding of the c-Maf-driven Treg/Tfr/microbiota interplay would be required prior to envisage c-Maf-targeted immunotherapy.

## Author Contributions

CI, HH, FA, and GV wrote the text and designed the figures.

### Conflict of Interest

The authors declare that the research was conducted in the absence of any commercial or financial relationships that could be construed as a potential conflict of interest.

## References

[1] Enzinger FM Shiraki M Musculo-aponeurotic fibromatosis of the shoulder girdle (extra-abdominal desmoid). Analysis of thirty cases followed up for ten or more years. Cancer. (1967) 20:1131–40. 6027003 10.1002/1097-0142(196707)20:7<1131::aid-cncr2820200716>3.0.co;2-8

[2] Nishizawa M Kataoka K Goto N Fujiwara KT Kawai S. v-maf, a viral oncogene that encodes a “Leucine Zipper” motif. Proc Natl Acad Sci USA. (1989) 86:7711–5. 10.1073/pnas.86.20.7711 2554284 PMC298140

[3] Kawai S Goto N Kataoka K Saegusa T Shinno-Kohno H Nishizawa M. Isolation of the avian transforming retrovirus, AS42, carrying the v-maf oncogene and initial characterization of its gene product. Virology. (1992) 188:778–84. 10.1016/0042-6822(92)90532-T 1585647

[4] Moussa WA Tadros MD Mekhael KG Darwish AE Shakir AH El-Rehim EA. Some simple methods of home processing and their implication with weaning foods. Nahrung. (1992) 36:26–33. 10.1002/food.19920360106 1579153

[5] Kawauchi S Takahashi S Nakajima O Ogino H Morita M Nishizawa M. Regulation of lens fiber cell differentiation by transcription factor c-Maf. J Biol Chem. (1999) 274:19254–60. 10.1074/jbc.274.27.19254 10383433

[6] Ring BZ Cordes SP Overbeek PA Barsh GS. Regulation of mouse lens fiber cell development and differentiation by the Maf gene. Development. (2000) 127:307–17. 10603348 10.1242/dev.127.2.307

[7] Kim JI Li T Ho IC Grusby MJ Glimcher LH. Requirement for the c-Maf transcription factor in crystallin gene regulation and lens development. Proc Natl Acad Sci USA. (1999) 96:3781–5. 10.1073/pnas.96.7.3781 10097114 PMC22371

[8] Wende H Lechner SG Cheret C Bourane S Kolanczyk ME Pattyn A. The transcription factor c-Maf controls touch receptor development and function. Science. (2012) 335:1373–6. 10.1126/science.1214314 22345400

[9] Wende H Lechner SG Birchmeier C. The transcription factor c-Maf in sensory neuron development. Transcription. (2012) 3:285–9. 10.4161/trns.21809 22889842 PMC3630182

[10] Imaki J Tsuchiya K Mishima T Onodera H Kim JI Yoshida K. Developmental contribution of c-maf in the kidney: distribution and developmental study of c-maf mRNA in normal mice kidney and histological study of c-maf knockout mice kidney and liver. Biochem Biophys Res Commun. (2004) 320:1323–7. 10.1016/j.bbrc.2004.05.222 15249232

[11] Huang W Lu N Eberspaecher H De Crombrugghe B. A new long form of c-Maf cooperates with Sox9 to activate the type II collagen gene. J Biol Chem. (2002) 277:50668–75. 10.1074/jbc.M206544200 12381733

[12] Hong E di Cesare PE Haudenschild DR. Role of c-maf in chondrocyte differentiation: a review. Cartilage. (2011) 2:27–35. 10.1177/1947603510377464 26069566 PMC4300789

[13] MacLean HE Kim JI Glimcher MJ Wang J Kronenberg HM Glimcher LH. Absence of transcription factor c-maf causes abnormal terminal differentiation of hypertrophic chondrocytes during endochondral bone development. Dev Biol. (2003) 262:51–63. 10.1016/S0012-1606(03)00324-5 14512017

[14] Kusakabe M Hasegawa K Hamada M Nakamura M Ohsumi T Suzuki H. c-Maf plays a crucial role for the definitive erythropoiesis that accompanies erythroblastic island formation in the fetal liver. Blood. (2011) 118:1374–85. 10.1182/blood-2010-08-300400 21628412

[15] Tsuchiya M Taniguchi S Yasuda K Nitta K Maeda A Shigemoto M. Potential roles of large mafs in cell lineages and developing pancreas. Pancreas. (2006) 32:408–16. 10.1097/01.mpa.0000220867.64787.99 16670624

[16] Kataoka K Shioda S Ando K Sakagami K Handa H Yasuda K. Differentially expressed Maf family transcription factors, c-Maf and MafA, activate glucagon and insulin gene expression in pancreatic islet alpha- and beta-cells. J Mol Endocrinol. (2004) 32:9–20. 10.1677/jme.0.0320009 14765989

[17] Parker ME Barrera A Wheaton JD Zuberbuehler MK Allan DSJ Carlyle JR. c-Maf regulates the plasticity of group 3 innate lymphoid cells by restraining the type 1 program. J Exp Med. (2020) 217:e20191030. 10.1084/jem.20191030 31570496 PMC7037249

[18] Pokrovskii M Hall JA Ochayon DE Yi R Chaimowitz NS Seelamneni H. Characterization of transcriptional regulatory networks that promote and restrict identities and functions of intestinal innate lymphoid cells. Immunity. (2019) 51:185–97.e186. 10.1016/j.immuni.2019.06.001 31278058 PMC6863506

[19] Zuberbuehler MK Parker ME Wheaton JD Espinosa JR Salzler HR Park E The transcription factor c-Maf is essential for the commitment of IL-17-producing gammadelta T cells. Nat Immunol. (2019) 20:73–85. 10.1038/s41590-018-0274-0 30538336 PMC6294311

[20] Liu M Zhao X Ma Y Zhou Y Deng M Ma Y. Transcription factor c-Maf is essential for IL-10 gene expression in B cells. Scand J Immunol. (2018) 88:e12701. 10.1111/sji.12701 29974486

[21] Vinson C Acharya A Taparowsky EJ. Deciphering B-ZIP transcription factor interactions *in vitro* and *in vivo*. Biochim Biophys Acta. (2006) 1759:4–12. 10.1016/j.bbaexp.2005.12.005 16580748

[22] Kataoka K. Multiple mechanisms and functions of Maf transcription factors in the regulation of tissue-specific genes. J Biochem. (2007) 141:775–81. 10.1093/jb/mvm105 17569705

[23] Kerppola TK Curran T. A conserved region adjacent to the basic domain is required for recognition of an extended DNA binding site by Maf/Nrl family proteins. Oncogene. (1994) 9:3149–58. 7936637

[24] Kerppola TK Curran T. Maf and Nrl can bind to AP-1 sites and form heterodimers with Fos and Jun. Oncogene. (1994) 9:675–84. 8108109

[25] Kataoka K Fujiwara KT Noda M Nishizawa M MafB, a new Maf family transcription activator that can associate with Maf and Fos but not with Jun. Mol Cell Biol. (1994) 14:7581–91. 10.1128/MCB.14.11.7581 7935473 PMC359294

[26] Yoshida MC Nishizawa M Kataoka K Goto N Fujiwara KT Kawai S Localization of the human MAF protooncogene on chromosome 16 to bands q22-q23. (Abstract). Cytogenet Cell Genet. (1991) 58:2003.

[27] Jamieson RV. Domain disruption and mutation of the bZIP transcription factor, MAF, associated with cataract, ocular anterior segment dysgenesis and coloboma. Hum Mol Genet. (2002) 11:33–42. 10.1093/hmg/11.1.33 11772997

[28] Chesi M Bergsagel PL Shonukan OO Martelli ML Brents LA Chen T. Frequent dysregulation of the c-maf proto-oncogene at 16q23 by translocation to an Ig locus in multiple myeloma. Blood. (1998) 91:4457–63. 10.1182/blood.V91.12.4457.412k48_4457_4463 9616139

[29] Hurt EM Wiestner A Rosenwald A Shaffer AL Campo E Grogan T. Overexpression of c-maf is a frequent oncogenic event in multiple myeloma that promotes proliferation and pathological interactions with bone marrow stroma. Cancer Cell. (2004) 5:191–9. 10.1016/S1535-6108(04)00019-4 14998494

[30] Morito N Yoh K Fujioka Y Nakano T Shimohata H Hashimoto Y. Overexpression of c-Maf contributes to T-cell lymphoma in both mice and human. Cancer Res. (2006) 66:812–9. 10.1158/0008-5472.CAN-05-2154 16424013

[31] Murakami YI Yatabe Y Sakaguchi T Sasaki E Yamashita Y Morito N. C-Maf expression in angioimmunoblastic T-cell lymphoma. Am J Surg Pathol. (2007) 31:1695–702. 10.1097/PAS.0b013e318054dbcf 18059226

[32] Uhlen M Zhang C Lee S Sjöstedt E Fagerberg L Bidkhori G. A pathology atlas of the human cancer transcriptome. Science. (2017) 357:eaan2507. 10.1126/science.aan2507 28818916

[33] Bauquet AT Jin H Paterson AM Mitsdoerffer M Ho I-C Sharpe AH. The costimulatory molecule ICOS regulates the expression of c-Maf and IL-21 in the development of follicular T helper cells and TH-17 cells. Nat Immunol. (2009) 10:167–175. 10.1038/ni.1690 19098919 PMC2742982

[34] Kurata H Lee HJ O'Garra A Arai N. Ectopic expression of activated Stat6 induces the expression of Th2-specific cytokines and transcription factors in developing Th1 cells. Immunity. (1999) 11:677–88. 10.1016/S1074-7613(00)80142-9 10626890

[35] Yang Y Ochando J Yopp A Bromberg JS Ding Y. IL-6 plays a unique role in initiating c-Maf expression during early stage of CD4 T cell activation. J Immunol. (2005) 174:2720–9. 10.4049/jimmunol.174.5.2720 15728480

[36] Xu J Yang Y Qiu G Lal G Wu Z Levy DE. c-Maf regulates IL-10 expression during Th17 polarization. J Immunol. (2009) 182:6226–36. 10.4049/jimmunol.0900123 19414776 PMC2834209

[37] Apetoh L Quintana FJ Pot C Joller N Xiao S Kumar D. The aryl hydrocarbon receptor interacts with c-Maf to promote the differentiation of type 1 regulatory T cells induced by IL-27. Nat Immunol. (2010) 11:854–61. 10.1038/ni.1912 20676095 PMC2940320

[38] Xu M Pokrovskii M Ding Y Yi R Au C Harrison OJ. c-MAF-dependent regulatory T cells mediate immunological tolerance to a gut pathobiont. Nature. (2018) 554:373–7. 10.1038/nature25500 29414937 PMC5814346

[39] Rutz S Noubade R Eidenschenk C Ota N Zeng W Zheng Y. Transcription factor c-Maf mediates the TGF-beta-dependent suppression of IL-22 production in T(H)17 cells. Nat Immunol. (2011) 12:1238–45. 10.1038/ni.2134 22001828

[40] Ouyang W Löhning M Gao Z Assenmacher M Ranganath S Radbruch A. Stat6-independent GATA-3 autoactivation directs IL-4-independent Th2 development and commitment. Immunity. (2000) 12:27–37. 10.1016/S1074-7613(00)80156-9 10661403

[41] Mari N Hercor M Denanglaire S Leo O Andris F. The capacity of Th2 lymphocytes to deliver B-cell help requires expression of the transcription factor STAT3. Eur J Immunol. (2013) 43:1489–98. 10.1002/eji.201242938 23504518

[42] Coyle AJ Lehar S Lloyd C Tian J Delaney T Manning S. The CD28-related molecule ICOS is required for effective T cell-dependent immune responses. Immunity. (2000) 13:95–105. 10.1016/S1074-7613(00)00011-X 10933398

[43] Nurieva RI Duong J Kishikawa H Dianzani U Rojo JM Ho IC. Transcriptional regulation of Th2 differentiation by inducible costimulator. Immunity. (2003) 18:801–11. 10.1016/S1074-7613(03)00144-4 12818161

[44] Paulos CM Paulos CM Carpenito C Plesa G Suhoski MM Varela-rohena A. The inducible costimulator (ICOS) is critical for the development of human T H 17 cells. (2010) 2:55ra78. 10.1126/scitranslmed.3000448 20980695 PMC6282816

[45] Pot C Jin H Awasthi A Liu SM Lai C-Y Madan R. Cutting edge: IL-27 induces the transcription factor c-Maf, cytokine IL-21, and the costimulatory receptor ICOS that coordinately act together to promote differentiation of IL-10-producing Tr1 cells. J Immunol. (2009) 183:797–801. 10.4049/jimmunol.0901233 19570826 PMC2768608

[46] Hooper KM Kong W Ganea D. Prostaglandin E2 inhibits Tr1 cell differentiation through suppression of c-Maf. PLoS ONE. (2017) 12:e0179184. 10.1371/journal.pone.0179184 28604806 PMC5467903

[47] Ranzani V Rossetti G Panzeri I Arrigoni A Bonnal RJ Curti S. The long intergenic noncoding RNA landscape of human lymphocytes highlights the regulation of T cell differentiation by linc-MAF-4. Nat Immunol. (2015) 16:318–25. 10.1038/ni.3093 25621826 PMC4333215

[48] Zhang F Liu G Wei C Gao C Hao J. Linc-MAF-4 regulates Th1/Th2 differentiation and is associated with the pathogenesis of multiple sclerosis by targeting MAF. FASEB J. (2017) 31:519–25. 10.1096/fj.201600838R 27756768

[49] Rodriguez A Vigorito E Clare S Warren MV Couttet P Soond DR. Requirement of bic/microRNA-155 for normal immune function. Science. (2007) 316:608–11. 10.1126/science.1139253 17463290 PMC2610435

[50] Su W Hopkins S Nesser NK Sopher B Silvestroni A Ammanuel S. The p53 transcription factor modulates microglia behavior through microRNA-dependent regulation of c-Maf. J Immunol. (2014) 192:358–66. 10.4049/jimmunol.1301397 24319262 PMC4195583

[51] Tamgue O Gcanga L Ozturk M Whitehead L Pillay S Jacobs R. Differential targeting of c-Maf, Bach-1, and Elmo-1 by microRNA-143 and microRNA-365 promotes the intracellular growth of *Mycobacterium tuberculosis* in alternatively IL-4/IL-13 activated macrophages. Front Immunol. (2019) 10:421. 10.3389/fimmu.2019.00421 30941122 PMC6433885

[52] Janiszewska J Szaumkessel M Kostrzewska-Poczekaj M Bednarek K Paczkowska J Jackowska J. Global miRNA expression profiling identifies miR-1290 as novel potential oncomiR in laryngeal carcinoma. PLoS ONE. (2015) 10:e0144924. 10.1371/journal.pone.0144924 26694163 PMC4692263

[53] Blonska M Joo D Nurieva RI Zhao X Chiao P Sun SC. Activation of the transcription factor c-Maf in T cells is dependent on the CARMA1-IKKbeta signaling cascade. Sci Signal. (2013) 6:ra110. 10.1126/scisignal.2004273 24345681 PMC5762115

[54] Qiang YW Ye S Chen Y Buros AF Edmonson R van Rhee F. MAF protein mediates innate resistance to proteasome inhibition therapy in multiple myeloma. Blood. (2016) 128:2919–30. 10.1182/blood-2016-03-706077 27793878 PMC5179331

[55] Niceta M Stellacci E Gripp KW Zampino G Kousi M Anselmi M. Mutations impairing GSK3-mediated MAF phosphorylation cause cataract, deafness, intellectual disability, seizures, and a down syndrome-like facies. Am J Hum Genet. (2015) 96:816–25. 10.1016/j.ajhg.2015.03.001 25865493 PMC4570552

[56] Hill EV Ng TH Burton BR Oakley CM Malik K Wraith DC. Glycogen synthase kinase-3 controls IL-10 expression in CD4(+) effector T-cell subsets through epigenetic modification of the IL-10 promoter. Eur J Immunol. (2015) 45:1103–15. 10.1002/eji.201444661 25627813 PMC4405077

[57] Liu CC Lai CY Yen WF Lin YH Chang HH Tai TS. Reciprocal regulation of C-Maf tyrosine phosphorylation by Tec and Ptpn22. PLoS ONE. (2015) 10:e0127617. 10.1371/journal.pone.0127617 25993510 PMC4439128

[58] Leavenworth JW Ma X Mo YY Pauza ME. SUMO conjugation contributes to immune deviation in nonobese diabetic mice by suppressing c-Maf transactivation of IL-4. J Immunol. (2009) 183:1110–9. 10.4049/jimmunol.0803671 19553542 PMC2965337

[59] Lin BS Tsai PY Hsieh WY Tsao HW Liu MW Grenningloh R. SUMOylation attenuates c-Maf-dependent IL-4 expression. Eur J Immunol. (2010) 40:1174–84. 10.1002/eji.200939788 20127678

[60] Hsu CY Yeh LT Fu SH Chien MW Liu YW Miaw SC. SUMO-defective c-Maf preferentially transactivates Il21 to exacerbate autoimmune diabetes. J Clin Invest. (2018) 128:3779–93. 10.1172/JCI98786 30059018 PMC6118635

[61] Ciofani M Madar A Galan C Sellars M Mace K Pauli F. A validated regulatory network for Th17 cell specification. Cell. (2012) 151:289–303. 10.1016/j.cell.2012.09.016 23021777 PMC3503487

[62] Cao S Liu J Song L Ma X. The Protooncogene c-Maf is an essential transcription factor for IL-10 gene expression in macrophages. J Immunol. (2005) 174:3484–92. 10.4049/jimmunol.174.6.3484 15749884 PMC2955976

[63] Saraiva M Christensen JR Veldhoen M Murphy TL Murphy KM O'Garra A. Interleukin-10 production by Th1 cells requires interleukin-12-induced STAT4 transcription factor and ERK MAP kinase activation by high antigen dose. Immunity. (2009) 31:209–19. 10.1016/j.immuni.2009.05.012 19646904 PMC2791889

[64] Neumann C Heinrich F Neumann K Junghans V Mashreghi M-F Ahlers J. Role of Blimp-1 in programing Th effector cells into IL-10 producers. J Exp Med. (2014) 211:1807–19. 10.1084/jem.20131548 25073792 PMC4144744

[65] Ouyang W Rutz S Crellin NK Valdez PA Hymowitz SG. Regulation and functions of the IL-10 family of cytokines in inflammation and disease. Annu Rev Immunol. (2011) 29:71–109. 10.1146/annurev-immunol-031210-101312 21166540

[66] Rojas JM Avia M Martin V Sevilla N. IL-10: a multifunctional cytokine in viral infections. J Immunol Res. (2017) 2017:6104054. 10.1155/2017/6104054 28316998 PMC5337865

[67] Gabryšová L Alvarez-Martinez M Luisier R Cox LS Sodenkamp J Hosking C C-Maf controls immune responses by regulating disease-specific gene networks and repressing IL-2 in CD4+T cells article. Nat Immunol. (2018) 19:497–507. 10.1038/s41590-018-0083-5 29662170 PMC5988041

[68] Veldhoen M Hirota K Westendorf AM Buer J Dumoutier L Renauld JC. The aryl hydrocarbon receptor links TH17-cell-mediated autoimmunity to environmental toxins. Nature. (2008) 453:106–9. 10.1038/nature06881 18362914

[69] Chang HD Helbig C Tykocinski L Kreher S Koeck J Niesner U. Expression of IL-10 in Th memory lymphocytes is conditional on IL-12 or IL-4, unless the IL-10 gene is imprinted by GATA-3. Eur J Immunol. (2007) 37:807–17. 10.1002/eji.200636385 17304625

[70] Ahyi AN Chang HC Dent AL Nutt SL Kaplan MH. IFN regulatory factor 4 regulates the expression of a subset of Th2 cytokines. J Immunol. (2009) 183:1598–606. 10.4049/jimmunol.0803302 19592658 PMC2734910

[71] Ho IC Hodge MR Rooney JW Glimcher LH. The proto-oncogene c-maf is responsible for tissue-specific expression of interleukin-4. Cell. (1996) 85:973–83. 10.1016/S0092-8674(00)81299-4 8674125

[72] Kim JI Ho IC Grusby MJ Glimcher LH The transcription factor c-Maf controls the production of interleukin-4 but not other Th2 cytokines. Immunity. (1999) 10:745–51. 10.1016/S1074-7613(00)80073-4 10403649

[73] Hodge MR Chun HJ Rengarajan J Alt A Lieberson R Glimcher LH. NF-AT-driven interleukin-4 transcription potentiated by NIP45. Science. (1996) 274:1903–5. 10.1126/science.274.5294.1903 8943202

[74] Li B Tournier C Davis RJ Flavell RA. Regulation of IL-4 expression by the transcription factor JunB during T helper cell differentiation. Eur Mol Biol Org J. (1999) 18:420–32. 10.1093/emboj/18.2.420 9889198 PMC1171136

[75] Ho IC Lo D Glimcher LH. c-maf promotes T helper cell type 2 (Th2) and attenuates Th1 differentiation by both interleukin 4-dependent and -independent mechanisms. J Exp Med. (1998) 188:1859–66. 10.1084/jem.188.10.1859 9815263 PMC2212398

[76] Mitchell RE Hassan M Burton BR Britton G Hill EV Verhagen J. IL-4 enhances IL-10 production in Th1 cells: implications for Th1 and Th2 regulation. Sci Rep. (2017) 7:11315. 10.1038/s41598-017-11803-y 28900244 PMC5595963

[77] Djuretic IM Levanon D Negreanu V Groner Y Rao A Ansel KM. Transcription factors T-bet and Runx3 cooperate to activate Ifng and silence Il4 in T helper type 1 cells. Nat Immunol. (2007) 8:145–53. 10.1038/ni1424 17195845

[78] Vinuesa CG Linterman MA Yu D MacLennan IC Follicular helper T cells. Annu Rev Immunol. (2016) 34:335–68. 10.1146/annurev-immunol-041015-055605 26907215

[79] Johnston RJ Poholek AC DiToro D Yusuf I Eto D Barnett B. Bcl6 and Blimp-1 are reciprocal and antagonistic regulators of T follicular helper cell differentiation. Science. (2009) 325:1006–10. 10.1126/science.1175870 19608860 PMC2766560

[80] Nurieva RI Chung Y Martinez GJ Yang XO Tanaka S Matskevitch TD. Bcl6 mediates the development of T follicular helper cells. Science. (2009) 325:1001–5. 10.1126/science.1176676 19628815 PMC2857334

[81] Leopold MF Begeman L van Bleijswijk JD LL IJ Witte HJ Grone A. Exposing the grey seal as a major predator of harbour porpoises. Proc Biol Sci. (2015) 282:20142429. 10.1098/rspb.2014.2429 25429021 PMC4262184

[82] Kroenke MA Eto D Locci M Cho M Davidson T Haddad EK. Bcl6 and Maf cooperate to instruct human follicular helper CD4 T cell differentiation. J Immunol. (2012) 188:3734–44. 10.4049/jimmunol.1103246 22427637 PMC3324673

[83] Andris F Denanglaire S Anciaux M Hercor M Hussein H Leo O. The transcription factor c-Maf promotes the differentiation of follicular helper T cells. Front Immunol. (2017) 8:480. 10.3389/fimmu.2017.00480 28496444 PMC5406410

[84] Eddahri F Denanglaire S Bureau F Spolski R Leonard WJ Leo O. Interleukin-6/STAT3 signaling regulates the ability of naive T cells to acquire B-cell help capacities. Blood. (2009) 113:2426–33. 10.1182/blood-2008-04-154682 19020307 PMC2656270

[85] Nurieva RI Mai XM Forbush K Bevan MJ Dong C. B7h is required for T cell activation, differentiation, and effector function. Proc Natl Acad Sci USA. (2003) 100:14163–8. 10.1073/pnas.2335041100 14615582 PMC283563

[86] Mak TW Shahinian A Yoshinaga SK Wakeham A Boucher LM Pintilie M. Costimulation through the inducible costimulator ligand is essential for both T helper and B cell functions in T cell-dependent B cell responses. Nat Immunol. (2003) 4:765–72. 10.1038/ni947 12833154

[87] Liu X Chen X Zhong B Wang A Wang X Chu F. Transcription factor achaete-scute homologue 2 initiates follicular T-helper-cell development. Nature. (2014) 507:513–8. 10.1038/nature12910 24463518 PMC4012617

[88] Liu X Lu H Chen T Nallaparaju KC Yan X Tanaka S. Genome-wide analysis identifies Bcl6-controlled regulatory networks during T follicular helper cell differentiation. Cell Rep. (2016) 14:1735–47. 10.1016/j.celrep.2016.01.038 26876184 PMC4975778

[89] Hiramatsu Y Suto A Kashiwakuma D Kanari H Kagami S-i Ikeda K. c-Maf activates the promoter and enhancer of the IL-21 gene, and TGF- inhibits c-Maf-induced IL-21 production in CD4+ T cells. J Leukoc Biol. (2010) 87:703–12. 10.1189/jlb.0909639 20042469

[90] Sahoo A Alekseev A Tanaka K Obertas L Lerman B Haymaker C. Batf is important for IL-4 expression in T follicular helper cells. Nat Commun. (2015) 6:7997. 10.1038/ncomms8997 26278622 PMC4557271

[91] Aschenbrenner D Foglierini M Jarrossay D Hu D Weiner HL Kuchroo VK. An immunoregulatory and tissue-residency program modulated by c-MAF in human TH17 cells. Nat Immunol. (2018) 19:1126–36. 10.1038/s41590-018-0200-5 30201991 PMC6402560

[92] Tanaka S Suto A Iwamoto T Kashiwakuma D Kagami S-i Suzuki K. Sox5 and c-Maf cooperatively induce Th17 cell differentiation via RORγt induction as downstream targets of Stat3. J Exp Med. (2014) 211:1857–74. 10.1084/jem.20130791 25073789 PMC4144730

[93] Sato K Miyoshi F Yokota K Araki Y Asanuma Y Akiyama Y. Marked induction of c-Maf protein during Th17 cell differentiation and its implication in memory Th cell development. J Biol Chem. (2011) 286:14963–71. 10.1074/jbc.M111.218867 21402704 PMC3083235

[94] Roncarolo MG Gregori S Battaglia M Bacchetta R Fleischhauer K Levings MK. Interleukin-10-secreting type 1 regulatory T cells in rodents and humans. Immunol Rev. (2006) 212:28–50. 10.1111/j.0105-2896.2006.00420.x 16903904

[95] Pot C Apetoh L Kuchroo VK. Type 1 regulatory T cells (Tr1) in autoimmunity. Semin Immunol. (2011) 23:202–8. 10.1016/j.smim.2011.07.005 21840222 PMC3178065

[96] Neumann C Blume J Roy U Teh PP Vasanthakumar A Beller A. c-Maf-dependent Treg cell control of intestinal TH17 cells and IgA establishes host-microbiota homeostasis. Nat Immunol. (2019) 20:471–81. 10.1038/s41590-019-0316-2 30778241

[97] Imbratta C Leblond MM Bouzourene H Speiser DE Velin D Verdeil G. Maf deficiency in T cells dysregulates Treg - TH17 balance leading to spontaneous colitis. Sci Rep. (2019) 9:6135. 10.1038/s41598-019-42486-2 30992496 PMC6468010

[98] Wheaton JD Yeh CH Ciofani M. Cutting edge: c-Maf is required for regulatory T cells to adopt RORgammat(+) and follicular phenotypes. J Immunol. (2017) 199:3931–6. 10.4049/jimmunol.1701134 29127150 PMC5728164

[99] Hussein HDS Van Gool F Azouz A Ajouaou Y El-Khatib H Oldenhove G. Multiple environmental signaling pathways control the differentiation of RORγt-expressing regulatory T cells. Front Immunol. (2019) 10:3007. 10.3389/fimmu.2019.03007 31998303 PMC6961548

[100] Panduro M Benoist C Mathis D. Tissue tregs. Annu Rev Immunol. (2016) 34:609–33. 10.1146/annurev-immunol-032712-095948 27168246 PMC4942112

[101] Ohnmacht C Park JH Cording S Wing JB Atarashi K Obata Y. Mucosal immunology. The microbiota regulates type 2 immunity through RORgammat(+) T cells. Science. (2015) 349:989–93. 10.1126/science.aac4263 26160380

[102] Lochner M Ohnmacht C Presley L Bruhns P Si-Tahar M Sawa S. Microbiota-induced tertiary lymphoid tissues aggravate inflammatory disease in the absence of RORgamma t and LTi cells. J Exp Med. (2011) 208:125–34. 10.1084/jem.20100052 21173107 PMC3023125

[103] Kühn R Löhler J Rennick D Rajewsky K Müller W. Interleukin-10-deficient mice develop chronic enterocolitis. Cell. (1993) 75:263–74. 10.1016/0092-8674(93)80068-P 8402911

[104] Roers A Siewe L Strittmatter E Deckert M Schlüter D Stenzel W T cell–specific inactivation of the interleukin 10 gene in mice results in enhanced T cell responses but normal innate responses to lipopolysaccharide or skin irritation. J Exp Med. (2004) 200:1289–97. 10.1084/jem.20041789 15534372 PMC2211912

[105] Huber S Gagliani N Esplugues E O'Connor W Jr Huber FJ Chaudhry A. Th17 cells express interleukin-10 receptor and are controlled by Foxp3(-) and Foxp3+ regulatory CD4+ T cells in an interleukin-10-dependent manner. Immunity. (2011) 34:554–65. 10.1016/j.immuni.2011.01.020 21511184 PMC3113617

[106] Rubtsov YP Rasmussen JP Chi EY Fontenot J Castelli L Ye X. Regulatory T cell-derived interleukin-10 limits inflammation at environmental interfaces. Immunity. (2008) 28:546–58. 10.1016/j.immuni.2008.02.017 18387831

[107] Chaudhry A Samstein RM Treuting P Liang Y Pils MC Heinrich J-m. Interleukin-10 signaling in regulatory T cells is required for suppression of Th17 cell-mediated inflammation. Immunity. (2011) 34:566–78. 10.1016/j.immuni.2011.03.018 21511185 PMC3088485

[108] Rescigno M. Tfr cells and IgA join forces to diversify the microbiota. Immunity. (2014) 41:9–11. 10.1016/j.immuni.2014.06.012 25035948

[109] Giordano M Henin C Maurizio J Imbratta C Bourdely P Buferne M. Molecular profiling of CD8 T cells in autochthonous melanoma identifies Maf as driver of exhaustion. EMBO J. (2015) 34:2042–58. 10.15252/embj.201490786 26139534 PMC4551351

[110] Speiser DE Ho PC Verdeil G. Regulatory circuits of T cell function in cancer. Nat Rev Immunol. (2016) 16:599–611. 10.1038/nri.2016.80 27526640

[111] Wherry EJ. T cell exhaustion. Nat Immunol. (2011) 12:492–9. 10.1038/ni.2035 21739672

[112] Chihara N Madi A Kondo T Zhang H Acharya N Singer M. Induction and transcriptional regulation of the co-inhibitory gene module in T cells. Nature. (2018) 558:454–9. 10.1038/s41586-018-0206-z 29899446 PMC6130914

[113] Mielke LA Liao Y Clemens EB Firth MA Duckworth B Huang Q. TCF-1 limits the formation of Tc17 cells via repression of the MAF-RORgammat axis. J Exp Med. (2019) 216:1682–99. 10.1084/jem.20181778 31142588 PMC6605755

[114] Vivier E Artis D Colonna M Diefenbach A Di Santo JP Eberl G. Innate lymphoid cells: 10 years on. Cell. (2018) 174:1054–66. 10.1016/j.cell.2018.07.017 30142344

[115] Sawa S Lochner M Satoh-Takayama N Dulauroy S Berard M Kleinschek M. RORgammat+ innate lymphoid cells regulate intestinal homeostasis by integrating negative signals from the symbiotic microbiota. Nat Immunol. (2011) 12:320–6. 10.1038/ni.2002 21336274

[116] Harly C Cam M Kaye J Bhandoola A. Development and differentiation of early innate lymphoid progenitors. J Exp Med. (2018) 215:249–62. 10.1084/jem.20170832 29183988 PMC5748853

[117] Gury-BenAri M Thaiss CA Serafini N Winter DR Giladi A Lara-Astiaso D. The spectrum and regulatory landscape of intestinal innate lymphoid cells are shaped by the microbiome. Cell. (2016) 166:1231–46.e1213. 10.1016/j.cell.2016.07.043 27545347

[118] Colonna M. Innate lymphoid cells: diversity, plasticity, and unique functions in immunity. Immunity. (2018) 48:1104–17. 10.1016/j.immuni.2018.05.013 29924976 PMC6344351

[119] Song C Lee JS Gilfillan S Robinette ML Newberry RD Stappenbeck TS. Unique and redundant functions of NKp46+ ILC3s in models of intestinal inflammation. J Exp Med. (2015) 212:1869–82. 10.1084/jem.20151403 26458769 PMC4612098

[120] Buonocore S Ahern PP Uhlig HH Ivanov II Littman DR Maloy KJ. Innate lymphoid cells drive interleukin-23-dependent innate intestinal pathology. Nature. (2010) 464:1371–5. 10.1038/nature08949 20393462 PMC3796764

[121] Klose CS Kiss EA Schwierzeck V Ebert K Hoyler T d'Hargues Y. A T-bet gradient controls the fate and function of CCR6-RORgammat+ innate lymphoid cells. Nature. (2013) 494:261–5. 10.1038/nature11813 23334414

[122] Papotto PH Ribot JC Silva-Santos B. IL-17(+) gammadelta T cells as kick-starters of inflammation. Nat Immunol. (2017) 18:604–11. 10.1038/ni.3726 28518154

[123] Cua DJ Tato CM. Innate IL-17-producing cells: the sentinels of the immune system. Nat Rev Immunol. (2010) 10:479–89. 10.1038/nri2800 20559326

[124] Wang S Juan J Zhang Z Du Y Xu Y Tong J. Inhibition of the deubiquitinase USP5 leads to c-Maf protein degradation and myeloma cell apoptosis. Cell Death Dis. (2017) 8:e3058. 10.1038/cddis.2017.450 28933784 PMC5636991

[125] Chen XH Xu YJ Wang XG Lin P Cao BY Zeng YY. Mebendazole elicits potent antimyeloma activity by inhibiting the USP5/c-Maf axis. Acta Pharmacol Sin. (2019) 40:1568–77. 10.1038/s41401-019-0249-1 31197245 PMC7468578

[126] Pellegrino S Ronda L Annoni C Contini A Erba E Gelmi ML. Molecular insights into dimerization inhibition of c-Maf transcription factor. Biochim Biophys Acta. (2014) 1844:2108–15. 10.1016/j.bbapap.2014.09.003 25220806

[127] Mao X Stewart AK Hurren R Datti A Zhu X Zhu Y. A chemical biology screen identifies glucocorticoids that regulate c-maf expression by increasing its proteasomal degradation through up-regulation of ubiquitin. Blood. (2007) 110:4047–54. 10.1182/blood-2007-05-088666 17875808

[128] Sefik E Geva-Zatorsky N Oh S Konnikova L Zemmour D McGuire AM. Mucosal immunology. Individual intestinal symbionts induce a distinct population of RORgamma(+) regulatory T cells. Science. (2015) 349:993–7. 10.1126/science.aaa9420 26272906 PMC4700932

[129] Kim KS Hong SW Han D Yi J Jung J Yang BG. Dietary antigens limit mucosal immunity by inducing regulatory T cells in the small intestine. Science. (2016) 351:858–63. 10.1126/science.aac5560 26822607

